# One-Shot Learning for Partial Discharge Diagnosis Using Ultra-High-Frequency Sensor in Gas-Insulated Switchgear

**DOI:** 10.3390/s20195562

**Published:** 2020-09-28

**Authors:** Vo-Nguyen Tuyet-Doan, The-Duong Do, Ngoc-Diem Tran-Thi, Young-Woo Youn, Yong-Hwa Kim

**Affiliations:** 1 Department of Electronic Engineering, Myongji University, Yongin 17058, Korea; tuyetdoan201096@gmail.com (V.-N.T.-D.); theduong@mju.ac.kr (T.-D.D.); ngocdiem15051996@gmail.com (N.-D.T.-T.); 2 HVDC Research Division, Korea Electrotechnology Research Institute (KERI), Changwon 51543, Korea; ywyoun@keri.re.kr

**Keywords:** ultra-high-frequency (UHF) sensor, gas-insulated switchgear (GIS), partial discharges (PDs), fault diagnosis, one-shot learning, Siamese network

## Abstract

In recent years, deep learning has been successfully used in order to classify partial discharges (PDs) for assessing the condition of insulation systems in different electrical equipment. However, fault diagnosis using deep learning is still challenging, as it requires a large amount of training data, which is difficult and expensive to obtain in the real world. This paper proposes a novel one-shot learning method for fault diagnosis using a small dataset of phase-resolved PDs (PRPDs) in a gas-insulated switchgear (GIS). The proposed method is based on a Siamese network framework, which employs a distance metric function for predicting sample pairs from the same PRPD class or different PRPD classes. Experimental results over the small PRPD dataset that was obtained from an ultra-high-frequency sensor in the GIS show that the proposed method achieves outstanding performance for PRPD fault diagnosis as compared with the previous methods.

## 1. Introduction

Power systems are being increasingly employed due to the increase in the demand for electricity, and their stability is important for stable operation of the power grid [1]. A gas-insulated switchgear (GIS) applied to substations is a major protection device for power facilities. GIS protects the power system by blocking excessive current quickly in the case of failure as well as normal opening and closing. When a failure occurs in a GIS, the impact of the accident is severe; hence, recovery takes a considerable amount of time and the power outage time increases. Various faults that cause an insulation breakdown of a GIS can be detected while using partial discharges (PDs) before insulation breakdown [2]. Therefore, detecting PDs in a GIS is necessary to ensure the safety and reliability of grid assets [3].

Electrical, mechanical, and chemical methods have been used in order to detect PDs in GISs [4,5,6,7]. Dissolved-gas analysis was employed in the chemical methods and ultra-high-frequency (UHF) sensors were used in the electromagnetic methods to detect PDs for on-site PD monitoring [8,9]. UHF sensors have the advantages of external interference immunity and high-sensitivity detection [10,11]. In this study, a UHF sensor was utilized for the PD measurement system [12].

Time-resolved PD (TRPD) and phase-resolved PD (PRPD) were used to investigate the PD characteristics in a GIS [13,14,15]. The TRPD-based methods analyze the PD pulses while using time-domain, frequency-domain, and both time- and frequency-domain features [16,17,18]. The PRPD-based methods analyze the phase-amplitude-number (ϕ-*q*-*n*) of the PRPDs, where ϕ is the phase angle, *q* is the amplitude, and *n* is the number of PD occurrences [19]. The defect types were identified by analyzing the number of PD pulses, and the maximum amplitude or average amplitude in each phase [20]. Using the features from the PRPDs, machine-learning-based classifiers, such as support vector machines (SVMs) [21], decision trees [22], and neural networks [23,24], were proposed for PD classification.

Deep neural networks, which combine feature extraction and classification, have achieved promising results in several application areas, such as computer vision, natural language processing, speech recognition, and text classification [25]. Deep learning models, such as convolutional neural networks (CNNs) [26], recurrent neural networks (RNNs) [27], and self-attention [28], have been employed and state-of-the-art results have been obtained, in order to improve the performance for fault diagnosis while using a PRPD in a GIS. [26] proposed a CNN to learn the local response from the temporal or spatial signals of a PRPD. [27] proposed an RNN model with a long short-term memory to process sequential PRPD data. To overcome parallel computation and enable the capture of the interactions among PRPDs, [28] used a self-attention neural network to assess the possibility of focusing on important information and simultaneous computation from PRPD input. However, most of the existing deep-learning-based fault diagnosis methods require a large dataset for training and validation [26,27,28]. However, it is difficult to obtain a large dataset for fault diagnosis in the real world [27,28].

In this paper, we propose a new one-shot learning model for fault diagnosis with a small number of PRPDs while using Siameses neural networks that have the advantage of reducing the parameters to train and avoiding the problem of over-fitting [29]. One-shot learning and few-shot learning can learn features when only a few labeled samples are provided [30]. The proposed method uses a Siamese structure consisting of two identical CNNs and a distance metric function [31]. The two CNNs share the same parameters and map the PRPDs into a suitable embedding space. Subsequently, the distance metric function calculates the distance between the two CNN outputs. During the training phase, two PRPDs from the same class or different classes are paired and the paired sample is processed through the Siamese network to train the model for binary classification. The proposed model detects faults in the GIS while using the test PRPD pair and the support set from the training data. The experimental results with a small number of PRPDs show that the proposed one-shot learning model demonstrates a better performance for the PRPD classification than a CNN and linear SVM. The main contributions of this paper are summarized, as follows:

- One-shot learning is introduced for the first time to classify the PRPDs in a GIS. This method offers the advantages of a high classification accuracy while requiring a small amount of data compared with a linear SVM and CNN [30]. The proposed model uses pairs of samples of the same class or different classes during the training phase and recognizes the test sample with a single training sample for each class.
- The proposed model uses a distance metric function to map the PRPDs into a suitable embedding space and predicts the test PRPD class conditioned on the distance, which improves the classification performance as compared with that of the CNN [30].
- The proposed model is verified through PRPD and on-site noise measurements using a UHF sensor. The proposed model achieves a classification accuracy of 98.65% for four types of faults and noise in the GIS.

The remainder of this paper is organized, as follows: the PRPD and noise measurements in the GIS are introduced in Section 2. Section 3 presents the architecture of the proposed one-shot learning model. We compare the performance of the proposed method with that of other conventional methods in Section 4. Finally, we conclude this paper in Section 5.

## 2. Prpd and Noise Measurements

**Figure 1:**
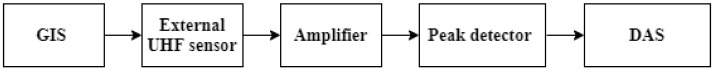
Block diagram of the partial discharge (PD) monitoring system.

In this section, we present the PRPD and noise measurements that were obtained using a PD monitoring system for the GIS [27,28]. Figure 1 shows a block diagram of the PD monitoring system, consisting of a GIS, external UHF sensor, amplifier, a peak detector, and a data acquisition system (DAS). A cavity-backed patch antenna is used as the external UHF sensor, the amplifier has a gain of 45 dB, and the operating bandwidth is between 500 MHz and 1.5 GHz [27,28]. The peak detector is used to capture maximum values of UHF PD pulses [32]. After the peak detector, the DAS uses an analog-to-digital converter (ADC) with $1024\times f_{m}$ samples per second, where $f_{m}=60$ Hz is the power frequency. Subsequently, the maximum value is captured at every 8 samples in the DAS and P=128 samples in each power cycle are used for PRPD measurements. The DAS uses an eight-bit analog-to-digital converter (ADC) with $P\times f_{m}$ samples per second, where P=128 is the number of data points in each power cycle and $f_{m}=60$ Hz is the power frequency. The measured signal at the *p*-th data point for the *m*-th power cycle is defined as x(m,p)∈{0,1,⋯,255}. Subsequently, the measured signal for M=3600 power cycles is defined in a matrix form, as

(1) $$X=\left[\begin{array}{cccc} x(1,1) & x(1,2) & \ldots & x(1,P)\\ x(2,1) & x(2,2) & \ldots & x(2,P)\\ \vdots & \vdots & \ddots & \vdots \\ x(M,1) & x(M,2) & \ldots & x(M,P) \end{array}\right].$$

### 2.1. Prpd Measurements in Gis

**Figure 2:**
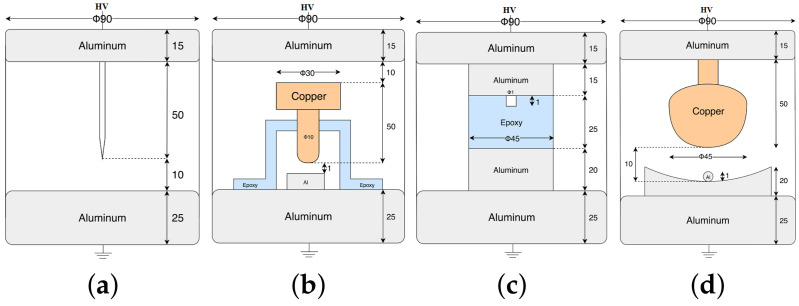
Artificial cells for the simulated (**a**) corona, (**b**) floating, (**c**) void, and (**d**) particle PDs.

For the PRPDs in the GIS, we investigate four types of faults, namely, protruding electrodes, floating electrodes, void defects, and free particles, whlie using artificial cells [27,28]. Figure 2 shows four artificial cells used to simulate the defects in GISs [27,28], where an artificial cell is built for each fault. This is because failure in GIS occurs very rarely in on-site environments. For corona, the artificial cell simulated with a sharp protrusion fixed on an electrode caused a local electric field enhancement through a needle, with a tip radius of 10 μm and a diameter of 1 mm. The distance between the needle and the ground electrode was 10 mm, and the test voltage was 11 kV, as displayed in Figure 2a. To simulate an unconnected cell with a test voltage of 10 kV, Figure 2b shows the cell of a fabricated floating electrode (with the distances of 10 mm between the high-voltage (HV) and middle electrodes and 1 mm between the middle and ground electrodes). Small voids between the epoxy disc and the upper electrode were formed to simulate the artificial void discharge, at the test voltage of 8 kV, as shown in Figure 2c. Figure 2d simulates the free particle discharge with a test voltage of 10 kV. A small sphere with a diameter of 1 mm was placed on a concave ground electrode and the HV electrode was attached to a sphere of diameter 45 mm (fixed at 10 mm from the ground electrode). The artificial cells of the four faults were filled with 0.2 MPa of sulfur hexafluoride (SF${}_{6}$) gas and the experiments under each failure are completed at one time.

**Figure 3:**
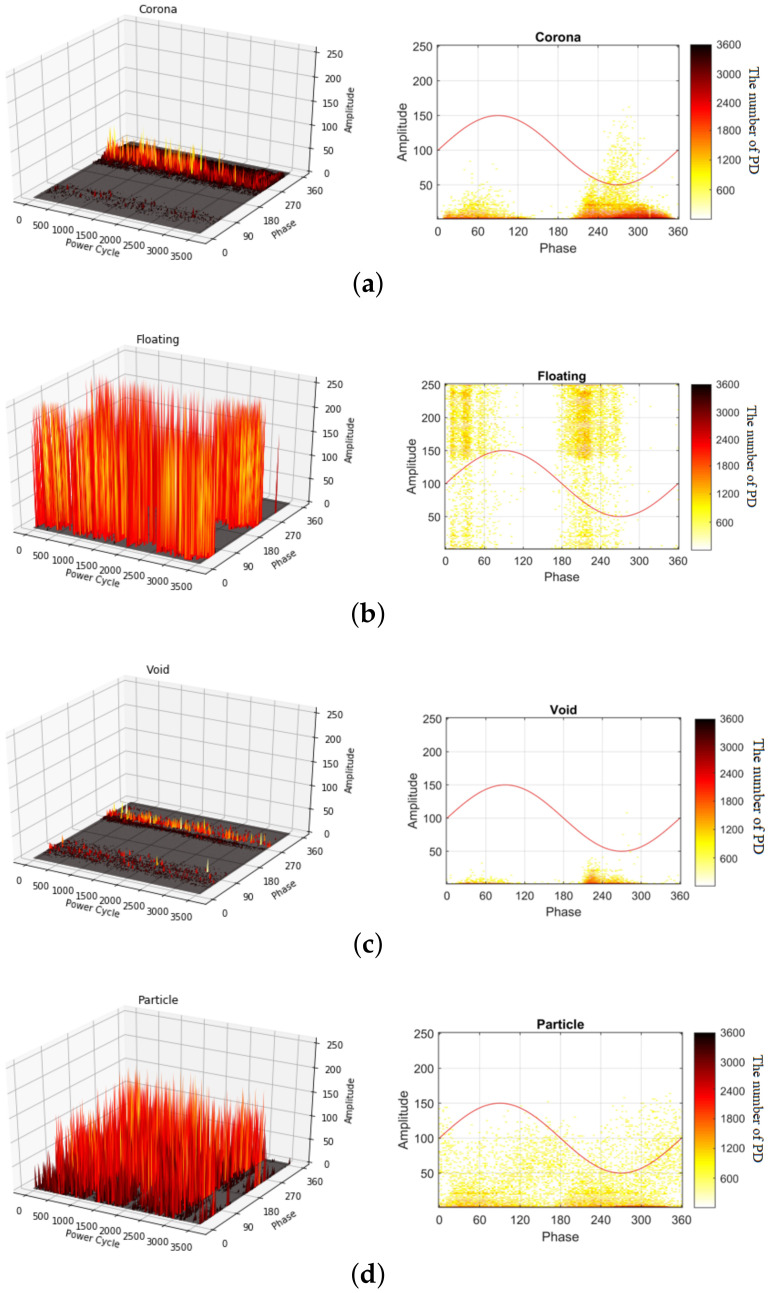
Examples of PRPDs in 3D and 2D representations: (**a**) corona, (**b**) floating, (**c**) void, and (**d**) particle faults.

Figure 3 shows the PRPDs for the four types of faults in three-dimensional (3D) and two-dimensional (2D) representations, where the amplitudes of the PRPDs for M=3600 power cycles are accumulated to generate the 2D PRPD patterns, and the number of PD events per M=3600 power cycles is represented by different colors. The measured signals for the corona fault exist on the negative half-cycle of the AC sine wave (from $180^{\circ }$ to $360^{\circ }$). At approximately $45^{\circ }$ of the cycle, the signal is low and close to zero. The floating PDs are presented during the first half of the positive and negative cycles of the AC sine wave with an extremely high signal density. The measured signals for the void fault occur in the $45^{\circ }$–$90^{\circ }$ and $180^{\circ }$–$270^{\circ }$ phases with low amplitude. For the particle fault, a PD signal of relatively high amplitude is distributed across all of the phases.

**Figure 4:**
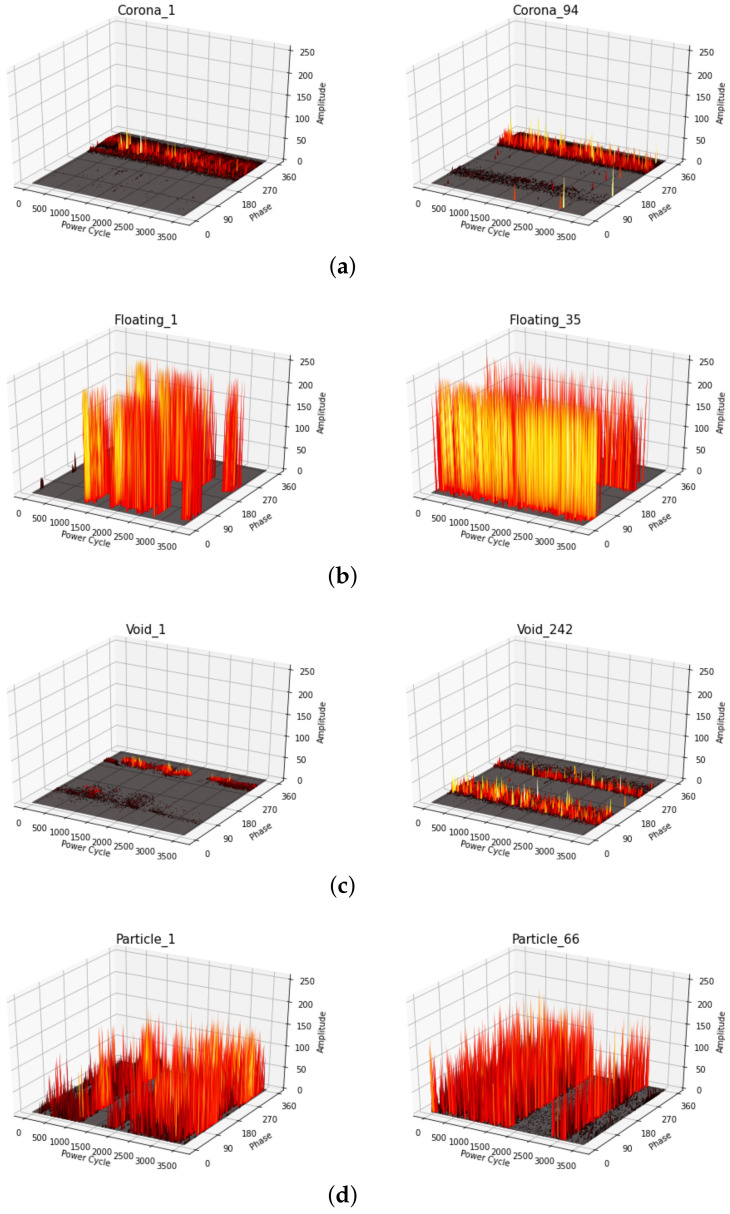
Discharge characteristics for the beginning (**left**) and the end (**right**) of PRPD measurements: (**a**) corona, (**b**) floating, (**c**) void, and (**d**) particle faults.

Figure 4 shows changes in discharge characteristics with the duration of PRPD measurements. There are similar patterns between the beginning and the end of PRPDs since PRPD measurements were performed for tens of minutes.

### 2.2. On-Site Noise Measurements

**Figure 5:**
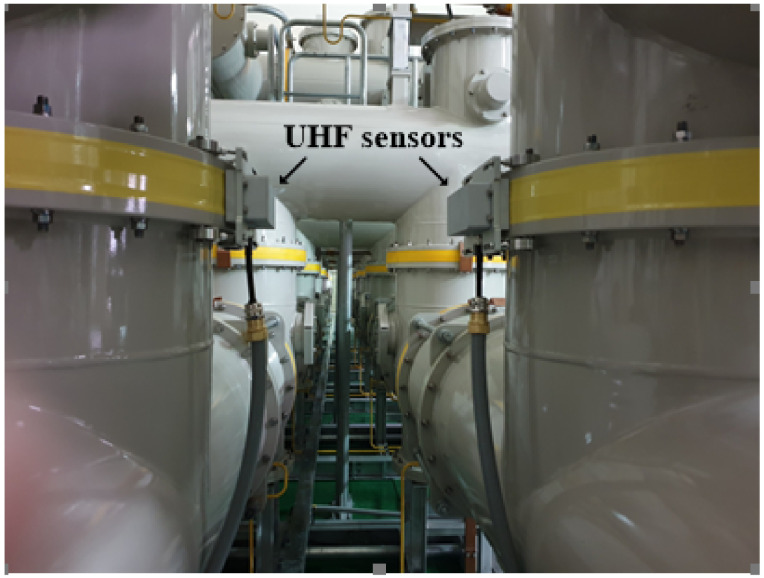
On-site noise measurements.

**Figure 6:**
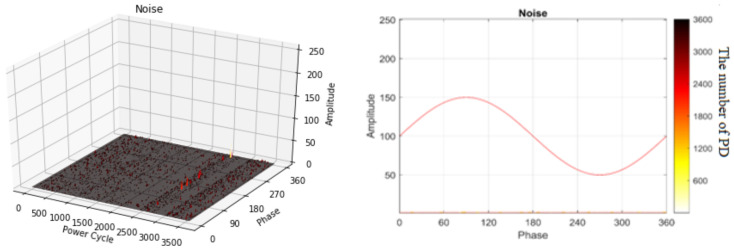
Example of on-site noise measurements in three-dimensional (3D) and two-dimensional (2D) representations.

On-site noise was measured for 267 min. in a field in Korea [28]. Figure 5 shows the on-site noise measurements in the GIS, where the external UHF sensor is located on the outside of the spacer in the GIS. Figure 6 shows an example of the on-site noise signal measurement in 3D and 2D representations. External noise signals appear across all of the phases in each power cycle and they are smaller than the PRPD signals in the GIS. In this paper, we regard on-site noise as a normal state for classification.

## 3. Proposed Method

**Figure 7:**
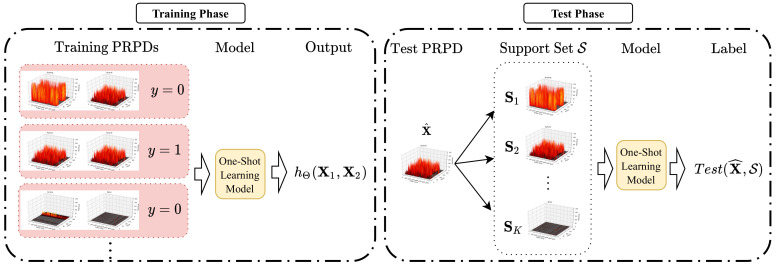
Architecture of the proposed one-shot learning model.

The proposed model for PRPD fault diagnosis is based on the architecture of one-shot learning [33]. Figure 7 shows the architecture of the proposed one-shot learning model, where the dataset is divided into three parts, namely, a training set T, test set $\hat{T}$, and support set S. The training set T is the collection of sample pairs from the same class or different classes. In the test phase, we denote a scenario with the support set S consisting of *K* labels with *N* samples per class as *K*-way *N*-shot classification. Here, we consider that one PRPD (N=1) is provided for each class as a sample for the support set S, i.e., *K*-way one-shot classification for PRPD fault diagnosis.

### 3.1. One-Shot Learning Model

**Figure 8:**
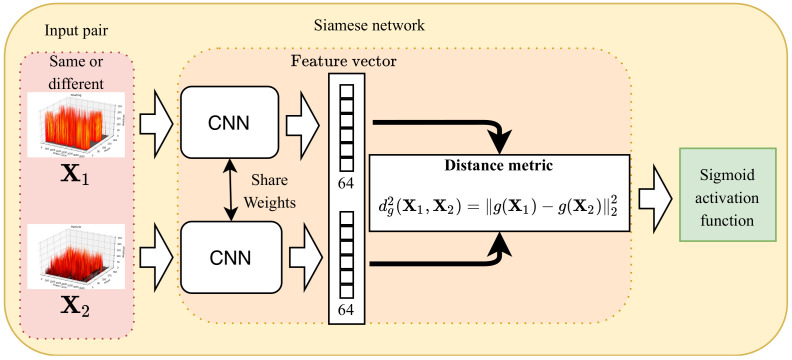
One-shot learning model using a Siamese network.

Figure 8 shows the proposed one-shot learning model for PRPD fault diagnosis, which is based on a Siamese neural network [31]. The Siamese neural network is composed of an input layer, two identical CNNs, a distance metric, and an output layer.

In the input layer, we denote $T=(X_{1},X_{2})\in T$ as an input pair of the same fault class or different fault classes, where $X_{1}$ and $X_{2}$ are the two PRPD signals in (1), respectively.

The same parameters and weights are used for the two identical CNNs, which are connected by a distance metric. Each CNN is composed of convolutional, max-pooling, dropout, flatten, and fully connected layers. The convolutional layers with multiple filters use the rectified linear unit (ReLU) activation function to improve the speed of the backpropagation computation and reduce the gradient-disappearance probability [34]. The max-pooling layer is used in order to reduce the number of computations and optimize the calculation space. Furthermore, dropout is added for regularization [35] and batch normalization is utilized in order to speed up learning by normalizing the input of the convolutional layers [36]. Flatten is used to transform a two-dimensional matrix into a vector. The distance metric is calculated based on the L2 norm as

(2) $$d_{g}^{2}\left(X_{1},X_{2}\right)={∥g\left(X_{1}\right)-g\left(X_{2}\right)∥}_{2}^{2},$$

where $g(X_{1})$ and $g(X_{2})$ are the feature vectors that are extracted by the CNN from $X_{1}$ and $X_{2}$, respectively, and g(·) is the CNN.

In the proposed one-shot learning model, the output represents the probability of showing the similarity or dissimilarity between two input samples and it is related to the distance metric, as follows:

(3) $$h_{\Theta }\left(X_{1},X_{2}\right)=\sigma \left(\alpha d_{g}^{2}\left(X_{1},X_{2}\right)+b\right)),$$

where σ(·) is the sigmoid function, α is a weight value, and *b* is a bias. In addition, Θ is denoted as a vector containing every parameter in the model that should be determined.

### 3.2. Network Optimization

Figure 7 shows the one-shot learning training for PRPD fault diagnosis. For the training phase, we denote *y* as the label that corresponds to $T=(X_{1},X_{2})\in T$, where y=1 if both $X_{1}$ and $X_{2}$ are from the same class, and y=0, otherwise. From the PRPD measurement data, we create the set of input and output pairs (T,y) to train and verify the proposed one-shot learning model. The parameters of the proposed model were learnt through the mini-batch B in order to minimize the following loss function:

(4) $$J\left(\Theta \right)=\frac{1}{\left|B\right|}\sum_{b\;\in B}Loss\left(b\right)+\frac{\lambda }{2|B|}{∥\Theta ∥}_{2}^{2},$$

where |·| is the number of elements in a set and λ is the parameter for the L2-norm regularization. In (4), the loss for the *b*-th input and output pair is calculated based on cross entropy, as

(5) $$Loss\left(b\right)=-y^{\left(b\right)}\log h_{\Theta }\left(X_{1}^{\left(b\right)},X_{2}^{\left(b\right)}\right)-\left(1-y^{\left(b\right)}\right)\log\left(1-h_{\Theta }\left(X_{1}^{\left(b\right)},X_{2}^{\left(b\right)}\right)\right),$$

where the superscript (b) is used to indicate the index of the *b*-th input and output pair for the mini-batch B.

Gradient descent is used to optimize neural networks and to alter the learning rate adaptively for minimizing the loss function. Numerous variations of the gradient descent method have been studied in previous studies e.g., AdaGrad, AdaDelta, Nesterov momentum into the ADAM, and Adam optimizer [37,38,39,40]. We select Adam as the optimizer with an initial learning rate of $6\times 10^{-5}$ [40].

In the testing phase, as shown in Figure 7, the support set $S=\{S_{1},\ldots ,S_{K}\}$ contains *K* PRPD training samples, where S⊂T and each sample in the support set corresponds to each class. Subsequently, the test sample $\hat{X}\in \hat{T}$ and each element $S_{k}$ in the support set S are entered into the Siamese network in order to calculate the similarity between the two inputs, where k=1,…,K. The label for the test sample $\hat{X}\in \hat{T}$ is determined based on the greatest similarity as

(6) $$Test\left(\hat{X},S\right)=\underset{k}{\arg\max}\left(h_{\Theta }\left(\hat{X},S_{k}\right)\right),$$

where the test set $\hat{T}$ and the support set S share the same label space. Finally, the accuracy of the proposed method is computed as

(7) $$\mathrm{Accuracy}=\frac{\mathrm{Number}\;\mathrm{of}\;Test(\hat{X},S)\;\mathrm{is}\;\mathrm{correctly}\;\mathrm{classified}}{\left|\hat{T}\right|}\times 100\%.$$

## 4. Experiment Results

We conducted PRPD experiments and noise measurements in the GIS in order to clarify the results of the one-shot learning algorithm for PRPD fault diagnosis. Table 1 shows the number of experiments for each fault, where the four PRPD faults, namely, corona, floating, particle, and void faults and noise, are coded by 0, 1, 2, 3, and 4, respectively. Each fault type of the PRPD signals and noise signals contains M=3600 power cycles and each power cycle has P=128 data points for one experiment.

In our experiments, we use 81% samples of the data as the training set T, 9% for the validation set V, and the remaining as the test set $\hat{T}$, where |T|=594, |V|=67, and $|\hat{T}|=74$, all of these sets are separate that have not each other appearing in its other stages. This has the advantage of demonstrating that the trained model does not have overfitting problems. We conducted extensive experiments to obtain hyperparameters for the different parameters used to tune our model. Some hyperparameters, such as the batch size, number of epochs, number of layers, kernel size, and number of kernels, were optimized. Table 2 illustrates the details of the CNN model in the proposed one-shot learning model. Moreover, we repeated the training process 10 times during our experiment in order to deal with the random initialization of the initial values for the training. The accuracy of the results was then averaged to confirm the validity of our model. All of the experiments were implemented based on TensorFlow [41] and Keras [42].

Table 3 shows the accuracy of the proposed one-shot learning model as compared with that of a linear SVM and CNN. The SVM is a well-known simple machine learning algorithm and the CNN model uses the same structure as the Siamese network and a softmax function for multi-class classification. In our experiments, the linear SVM uses a feature vector for the maximum values at each phase to classify the faults in the GIS, while using the parameter C=1.0. The proposed one-shot learning model achieved a classification accuracy of 98.65% and it showed a performance improvement of 2.7% over the CNN. This is because the distance metric of the proposed one-shot learning model uses two identical CNNs to map the PRPDs into a suitable embedding space and reduces the intra-class variation to avoid misclassification. In addition, the proposed one-shot learning model and CNN showed better performances than the SVM. The SVM requires appropriate feature extraction to train the classifier. However, the proposed one-shot learning model achieved a high classification accuracy while using a CNN structure without the feature extraction stage. For the corona fault, the classification accuracy was 100% for SVM, CNN, and the proposed one-shot learning model. The SVM showed the lowest performances of 25% and 28.57% for the floating and particle faults, respectively. This is because small training samples were provided for the floating and particle faults. The proposed one-shot learning model could classify each type of fault in the GIS and showed one error case in the void fault.

We also conducted comparing the classification results with the balanced dataset by randomly selecting 35 samples for each PRPD fault from the whole experimental dataset. It can be seen that the performance of the one-shot learning and CNN are 94.44%, 88.89%, respectively, which are all dramatically higher than 72.22%, the performance of SVM, as shown in Table 4. Besides that, the proposed one-shot learning method performs better by about 5.55% higher in accuracy than the CNN method.

**Figure 9:**
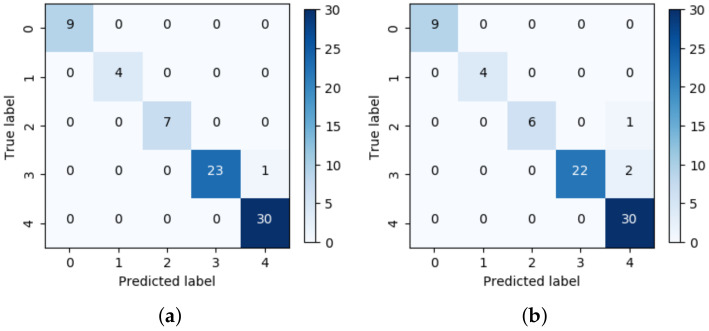
Confusion matrix with whole data case: (**a**) One-shot learning, (**b**) CNN.

Figure 9 shows the confusion matrix for the one-shot learning model and the CNN. The proposed one-shot learning model has one error case for the void fault and the CNN has three errors (one error for the particle fault and two errors for the void fault). All of the errors for the proposed one-shot learning model and CNN were misclassified as noise.

**Figure 10:**
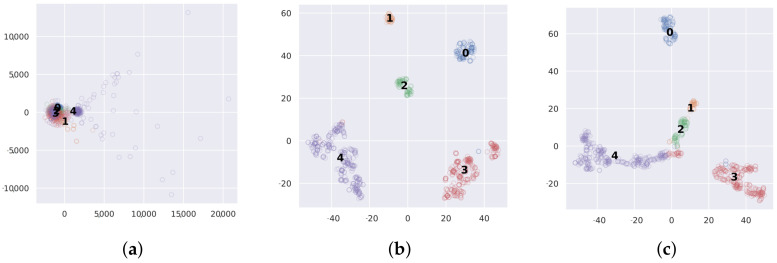
Visualization using t-SNE with whole data case: (**a**) Input, (**b**) One-shot learning, and (**c**) CNN.

The features of the proposed one-shot learning model were analyzed using t-distributed stochastic neighbor embedding (t-SNE) in order to understand the model learning better. Here, t-SNE reduces the dimensions of the data into 2D components with the maximum variation and visualizes them, such that similar features are transformed into nearby points. Figure 10 shows the t-SNE representations to visualize a set of inputs and their outputs of the one-shot learning model and the CNN, where the last fully connected layers g(X) in the Siamese network and the CNN are used. Figure 10a shows that numerous fault data are very close to the noise and, hence, are difficult to classify accurately using the input PRPDs. Figure 10b shows that the features of each fault for the one-shot learning model are separately distributed. As shown in Figure 10c, the particle and void faults slightly overlap with the noise sample using the CNN and, hence, the CNN has some errors in the particle and void faults.

## 5. Conclusions

In this study, we classified the PRPDs in a GIS while using a small amount of training data. Because fault diagnosis using deep learning will not fit well with small data, this paper addresses the challenge by adopting the Siamese network for one-shot learning. First, we used an external UHF sensor to acquire PRPD measurements from artificial cells and on-site noise in the GIS. Subsequently, we proposed a one-shot learning method based on a Siamese neural network framework. The proposed method extracts PRPD features from two identical CNNs, measures the distance between the PRPD features, and predicts whether their output pair is considered to be from the same class or different classes. Finally, we compare the experimental results for dataset cases: whole data and balanced data. The experimental results showed that the performance of the proposed method for PRPD classification with a small dataset was better than that of the SVM and CNN.

For future studies, we intend to design artificial cells of surface/creeping discharges on the surface of the GIS insulator for analyzing the PRPD patterns and conduct further verification of the proposed method for on-site fault data. 

## Figures and Tables

**Table 1:** Experimental dataset.

Fault Types	Corona	Floating	Particle	Void	Noise
	(0)	(1)	(2)	(3)	(4)
Number of experiments	94	35	66	242	298

**Table 2:** Details of the convolutional neural networks (CNN) in the proposed one-shot learning model.

		Kernel	Kernel		
No.	Layer Type	Size/Stride	Number	Output Size	Padding
1	Convolution 1	16 × 16/4	16	900 × 32 × 16	same
2	Max-Pooling 1	2 × 2/2	-	450 × 16 × 16	valid
3	Batch Normalization 1	-	-	450 × 16 × 16	-
4	Drop Out 1	-	-	450 × 16 × 16	-
5	Convolution 2	3 × 3/1	32	450 × 16 × 32	same
6	Max-Pooling 2	2 × 2/2	-	225 × 8 × 32	valid
7	Batch Normalization 2	-	-	225 × 8 × 32	-
8	Drop Out 2	-	-	225 × 8 × 32	-
9	Convolution 3	3 × 3/1	64	225 × 8 × 64	same
10	Max-Pooling 3	2 × 2/2	-	112 × 4 × 64	valid
11	Batch Normalization 3	-	-	112 × 4 × 64	-
12	Drop Out 3	-	-	112 × 4 × 64	-
13	Convolution 4	3 × 3/1	64	112 × 4 × 64	same
14	Max-Pooling 4	2 × 2/2	-	56 × 2 × 64	valid
15	Batch Normalization 4	-	-	56 × 2 × 64	-
16	Drop Out 4	-	-	56 × 2 × 64	-
17	Flatten 1	-	-	7168 × 1	-
18	Dense 2	64	-	64 × 1	-

**Table 3:** Performance comparisons in terms of accuracy.

Fault Types	Overall	Corona	Floating	Particle	Void	Noise
	(%)	(%)	(%)	(%)	(%)	(%)
**Linear SVM**	**92.28**	100	25	28.57	83.33	73.33
**CNN**	**95.95**	100	100	85.71	91.67	100
**One-shot learning**	**98.65**	100	100	100	95.83	100

**Table 4:** Performance comparisons with balanced data case.

Fault Types	Overall	Corona	Floating	Particle	Void	Noise
	(%)	(%)	(%)	(%)	(%)	(%)
**Linear SVM**	**72.22**	75	100	50	33.33	100
**CNN**	**88.89**	75	67.67	100	100	100
**One-shot learning**	**94.44**	75	100	100	100	100
